# Association Between Atherogenic, Thrombogenic, and Lipophilic Indices and the Odds of Diabetic Nephropathy in Type 2 Diabetic Patients: A Case–Control Study

**DOI:** 10.1002/fsn3.4686

**Published:** 2025-03-04

**Authors:** Behnood Abbasi, Elham Ghanbarzadeh, Bita Panahizadeh

**Affiliations:** ^1^ Department of Nutrition, Electronic Health and Statistics Surveillance Research Center, Science and Research Branch Islamic Azad University Tehran Iran; ^2^ Department of Nutrition, Science and Research Branch, Islamic Azad University Tehran Iran

**Keywords:** Atherogenic Index, Diabetic Nephropathy, Lipophilic Index, Thrombogenic Index, Type 2 Diabetes

## Abstract

The quality of fats in the diet affects the development of chronic kidney disease. In this study, we aimed to understand the relationship between dietary fat markers and the risk of diabetic nephropathy (DN) in patients with Type 2 diabetes (T2DM).In this case retrospective study, 309 patients with T2DM (151 with DN, and 158 without DN) were included. A 147 item questionnaire measuring food frequency and the International Physical Activity Questionnaire (IPAQ) were used. Anthropometric indices, and biochemical factors were measured or recorded from the patient's files. Quantitative Insulin Sensitivity Check Index (QUICKI), Homeostatic Model Assessment for Insulin Resistance (HOMA‐IR), atherogenic, thrombogenic, and lipophilic indices were calculated. Modified nutritionist IV software and the USDA composition table were used. Odds ratios (OR) and 95% confidence intervals (CI) were obtained using a case–control design. Age, BMI, energy in Model 1 and physical activity, blood glucose, level of insulin, lipid profile, creatinine level, and CRP were adjusted as interventions in Model 2. Compared to control subjects, patients with diabetic nephropathy (DN) had a significantly higher body mass index (BMI), level of fasting insulin, fasting blood sugar (FBS), total cholesterol (TC), triglyceride (TG), low‐density lipoprotein cholesterol (LDL‐C) and creatinine as well as lower intakes of energy, carbohydrates, mono—and polyunsaturated fatty acids, fiber, and cholesterol compared to controls. After adjustment for possible confounders, patients in the highest quartile of the atherogenic (OR: 3.49, 95% CI: 1.65–7.41), thrombogenic (OR: 4.3, 95% CI: 1.86–8.72), and lipophilic (OR: 3.50, 95% CI: 1.62–7.52) indices had significantly higher odds of DN than those in the lowest quartile. There was a considerable relationship between higher dietary fat indices (including atherogenic, thrombogenic, and lipophilic indices) and a higher chance of developing nephropathy.

## Graphical abstract

This is the first investigation that examined the association between atherogenic (IA), thrombogenic (IT), and lipophilic (LT) indices ‐as fat intake indicators‐ and diabetic nephropathy (DN). This study found Type 2 diabetes mellitus (T2DM) patients in the highest quartile of atherogenic, thrombogenic, and lipophilic indices had a greater likelihood of developing DN. Also, higher values of IA, IT, and LT were associated with a higher probability of DN in T2DM patients.

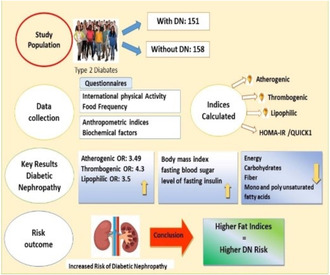

## 1 Introduction

Diabetes mellitus (DM) is a multifactorial disease that is influenced by genetic and environmental factors. (Artasensi, Pedretti, and Vistoli 2020), That negatively affects the absorption of carbohydrates, fats, and lipoproteins, leading to elevated blood sugar and various health issues, including hypertension, hyperinsulinemia, hyperlipidemia, and atherosclerosis. (Poznyak et al. 2020). By 2040, the number of people with diabetes is expected to hit 642 million, with studies suggesting that 90% will have Type 2 diabetes (T2DM). (Holman, Young, and Gadsby 2015; Zhang and Gregg 2017).

The 2019 Global Burden of Disease (GBD) study identified diabetes as the eighth leading cause of disability and death globally (GBD 2019). Diabetes imposes a significant burden on the health‐care system (Chan et al. 2021). The International Diabetes Federation (IDF) estimated the health costs of this disease. It is US$966 billion worldwide. In 2021, projected to exceed US$1054 billion by 2045 (International Diabetes Federation 2021).

T2DM is currently considered an inflammatory disease (Aktas, Atak, and Tel 2023). In addition, diabetes‐related outcomes such as atherosclerosis are also associated with inflammatory conditions (Sincer, Gunes, and Mansiroglu 2020). Recently, the CARE TIME study has shown that diabetic nephropathy (DN), one of the long‐term complications of diabetes caused by proteinuria and decreased Glomerular Filtration Rate (GFR) (Pelle et al. 2022), is associated with a higher inflammatory burden than diabetic without DN (Bilgin et al. 2021). Half of patients with T2DM and one‐third of T1DM patients will develop Diabetic kidney disease (DKD) during their lifetime (Sun et al. 2022).

A clinical diagnosis of DN can usually be made if microalbuminuria is detected in two out of three random urine samples collected in the past 6 months (excretion of albumin over 30 mg/g creatinine) (Association 2013). DN is characterized by glomerular hypertrophy, thickening of the basement and glomerular membranes, and accumulation of extracellular matrix in these membranes, leading to glomerular fibrosis and sclerosis (Lin, Wu, and Wang 2019). The pathophysiology of DN is influenced by several factors, including changes in metabolism and hemodynamics, blood sugar, lipid profile, the renin‐angiotensin system, and oxidative stress (Thipsawat 2021).

Treatment of DN is primarily aimed at preventing the progression of microalbuminuria to macroalbuminuria, which ultimately leads to reduced kidney function and cardiovascular disease. On the other hand, patients' nutritional status as a modifiable factor can influence DN processes and outcomes (Li et al. 2020; Liu et al. 2023). There are hybrid results regarding the effects of a high‐fat diet on the progress of chronic kidney complications. One study showed a negative association between PUFA intake and DKD in patients with T2DM, but no significant relationship between EPA and DHA and DKD. According to the data of this study, diets with PUFA are likely to have more beneficial clinical effects, and this relationship becomes stronger at higher levels of kidney disease. In this study, subjects with albuminuria reported significantly lower PUFA intake. It has also been reported that increased proteinuria in patients with Type 1 and Type 2 diabetes is positively associated with fatty acid intake and inversely associated with polyunsaturated fatty acid intake (Dos Santos et al. 2018). In contrast to the EPA and DHA study, it was shown that n‐3 PUFA reduces albuminuria (Lin et al. 2022). In a cross‐sectional study on 366 T2DM patients in Brazil, Multivariate analysis showed that the intake of linolenic acid and linoleic acid was negatively associated with DKD adjusted for sex, smoking, fasting plasma glucose, ACE inhibitors and/or angiotensin receptor blocker use, systolic blood pressure, cardiovascular disease, and HDL cholesterol (Dos Santos et al. 2018). However, more comprehensive studies are needed to provide dietary recommendations.

The Index of Atherogenicity (IA) reveals the relationship between the sum of SFAs and the sum of UFAs (Chen and Liu 2020). The main SFAs classes (C12:0, C14:0, and C16:0, except C18:0) are considered pro‐atherogenic. They enable lipid adhesion to the circulatory and immunological systems cells (Omri et al. 2019). The index of Thrombogenicity (IT) indicates blood clot formation tendency, and illustrates the relationship between anti‐thrombogenic fatty acids (MUFAs, Omega‐3 and 6 polyunsaturated fatty acids or PUFAs), and pro‐thrombogenic (C12:0, C14:0, and C16:0) ones (Khalili Tilami and Kouřimská 2022). The Lipophilic Index (LI) is a mean of fatty acid—in diet or adipose tissue—melting points correlated with higher carbon counts and greater saturation (Toledo et al. 2013). In addition, metabolic status is more affected by the type of fat consumed than by total fat intake (Shapiro et al. 2011). Attenuating endothelial dysfunction, reducing inflammation, and improving blood pressure control and dyslipidemia can effectively manage DKD (Kim et al. 2022). It has been shown PUFAs and MUFAs to be effective in reducing inflammation It has been shown that PUFAs and MUFAs to be effective in reducing inflammation (Poli, Agostoni, and Visioli 2023), and thus may affect DKD outcomes (Shapiro et al. 2011).

The association between a high‐fat diet and the risk of DN has not been reported. Therefore, this study aimed to evaluate the relationship between dietary fat intake indices, including atherogenic, thrombogenic, and lipophilic indices, and the risk of developing diabetic nephropathy in patients with Type 2 diabetes.

## 2 Methods and Materials

### 2.1 Participants

In this case–control retrospective study, patients with Type 2 diabetes who referred to the internal hospital were selected as the study population from August 2020 to May 2021. The case group consists only of patients with Type 2 diabetes aged between 18 and 60 years with nephropathy, criteria for exclusion consist of patients who were on special diets, failure to respond to more than 40 items in the food questionnaire, or extreme reported energy intake of less than 800 kcal or more than 4200 kcal. The control group was identical to those of the cases, except for the diabetic patients without nephropathy. Finally, 309 patients with Type 2 diabetes (151 patients with DN and 158 non‐DN) were enrolled.

### 2.2 Procedure

The purpose of the study was explained to the participants before registration and written consent was obtained. Then, sociodemographic information, physical activity, and the 147‐item semi quantitative food frequency questionnaire (SFFQ), whose reliability and validity were confirmed by Mirmiran et al. (2010), were completed for each participant by the researcher. Participants' physical activity measurements were based on the International Physical Activity Questionnaire (IPAQ) (Norman et al. 2002). The IPAQ questionnaire was developed by WHO and CDC in Geneva in 1998 for people aged 15–69 years and is a valid and reliable way to measure physical activity. There are two types of IPAQ questions: short and long. The short form (IPAQ‐S) was used in this study. The usual values of the modules and containers were explained to evaluate the food intake of each patient, and the daily intake of all foods was obtained from the food frequency questionnaires and converted to grams/day using household measurements.

### 2.3 Outcomes and Measurement

Body weight was measured to the nearest 0.1 kg using the Seca scale while participants were dressed. The weight (kg) /height (m^2^) ratio was used to compute body mass index (BMI). Biochemical variables such as fasting blood sugar (FBS), insulin level, C‐reactive protein (CRP), total cholesterol (TC), triglycerides (TG), high‐density lipoprotein cholesterol (HDL‐C), low‐density lipoprotein cholesterol (LDL‐C) and creatinine values were obtained from the participants' last 3‐month medical records. To estimate the intake of macronutrients, micronutrients, and energy, the modified nutritionist IV software for Iranian food and the USDA composition table were used to analyze the 147‐item SFFQ obtained.

The International Physical Activity Questionnaire (IPAQ) was used to calculate daily physical activity and was reported as metabolic equivalent (MET)‐minutes/week.

Quantitative Insulin Sensitivity Check Index (QUICKI) Homeostatic Model Assessment for Insulin Resistance (HOMA‐IR) (Muniyappa, Madan, and Varghese 2000), Index of Atherogenicity (IA), Index of Thrombogenicity (IT) (Chen and Liu 2020), and Lipophilic Index (LI) were calculated using the following appropriate formulas (Toledo et al. 2013).

$$\text{QUICKI}:1/\left[\mathrm{Log}\;\left(\text{Fasting Insulin},\mathrm{\mu U}/\mathrm{ml}\right)+\mathrm{Log}\;\left(\text{Fasting Glucose},\mathrm{mg}/\mathrm{dl}\right)\right]$$

$$\text{HOMA}-\mathrm{IR}:\left[\left(\text{Fasting Insulin}\;\left(\mathrm{\mu U}/\mathrm{mL}\right)\right)\times \left(\text{Fasting Glucose}\;\left(\text{mmol}/L\right)\right)\right]/22.5$$

The atherogenic index (AI) was modified in 1991 from the index developed by Ulbritcht and Southgate (1991) to characterize the atherogenic potential of FA. Because the PUFA/SFA ratio was too popular and unsuitable for assessing the atherogenicity of foods, Ulbricht and Southgate proposed a new index, AI, based mainly on PUFA/SFA, taking into account the available evidence and evaluated whether the values were appropriate. The formula for calculating IA is:

$$\mathrm{IA}=\left[C12:0+4\;\left(C14:0\right)+C16:0\right]/\left[\text{MUFA}+n-\text{3PUFA}+n-\text{6PUFA}\right].$$

The thrombosis index (IT) was created by Ulbricht and Southgate (1991) together with IA in 1991. The formula is:

$$\begin{array}{c} \mathrm{IT}=\left[C14:0+C16:0+C18:0\right]/[0.\text{5MUFA}+0.5\;\left(n-\text{6PUFA}\right)\\ \;+3\;\left(n-\text{3PUFA}\right)+\left(n-\text{3PUFA}/n-\text{6PUFA}\right)]. \end{array}$$

IT characterizes the thrombogenic potential of FA, indicates the need to form thrombi in blood vessels, and enables the involvement of various FAs that represent the relationship between mature thrombogenic FA (C12:0, C14:0, and C16:0) and the anti‐thrombogenic FAs (MUFAs and the n‐3 and n‐6 families) (Ulbricht and Southgate 1991). Therefore, food or food consumption may reduce usefulness which contributes to CVH. It has been used in many studies on fatty acids to assess the extent of thrombosis. As with the IA formulation, the proposed IT system ought to be changed as our knowledge of MUFA and trans fatty acids increases.

Dietary LI was calculated by adding the amount (in grams) of each fatty acid and its melting point (°C) (information on melting points obtained from the LipidBank database) divided by the number of fatty acids (Soltani et al. 2020).

$$\mathrm{LI}={Ʃ}_{k}\;\text{Fatty Acid}\;\left(g\right)\times \text{Melting Point}\;\left(c\right)/{Ʃ}_{k}\;\text{Fatty Acid}\;\left(g\right).$$

In this study, we obtained crude and adjusted odds ratios for three models; Crude, Model 1 by adjusting the confounders of age, BMI and energy, and Model 2 by adjusting the confounders of physical activity, blood glucose, insulin level, lipid profile, creatinine level and CRP.

### 2.4 Statistical Analysis

Gpower 3.1.9.2 software (Kiel University, Kiel, Germany) was used for calculating sample size (Erdfelder, Faul, and Buchner 1996), based on effect size = 0.15, *α* = 0.05, *β* = 0.05 (power = 0.95), and with 5° of freedom. The 138 patients were determined to be in each group. In this study, the attrition rate was estimated to be 10%, and thus, 158 participants were enrolled in each group (diabetic patients with and without nephropathy), seven patients with DN excluded from study and, finally, 309 patients with Type 2 diabetes (151 patients with DN and 158 non‐DN) were enrolled. Cases and controls just arbitrarily drawn from the patient pool and not paired.

Process of sample size calculation and power analysis:

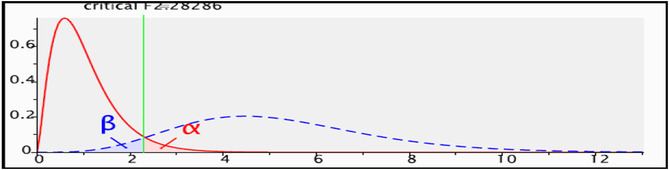

Using the G*Power software for the F tests—Linear multiple regression: Fixed model, *R* ^2^ deviation from zero.

**Analysis:** A priori: Compute required sample size.

**Input:** Effect size f^2^ = 0.15.

*α* err prob. = 0.05.

Power (1‐*β* err prob) = 0.95.

Number of predictors = 5.

**Output:** Noncentrality parameter λ = 20.7000000.

Critical *F* = 2.2828562.

Numerator df = 5.

Denominator df = 132.

Total sample size = 138.

Actual power = 0.9507643.

SPSS version 24 (SPSS Inc., Chicago, IL) was used. Visual examination of the histogram curve of data frequencies was used to assess the normality of data distribution. Continuous variables are presented as the mean ± standard deviation (SD), and qualitative data are reported as percentages and frequencies. Using independent sample *t*‐tests and analysis of covariance, continuous variables were compared between cases and controls. Categorical variables were compared using chi‐squared analysis. The odds ratio (OR) with a 95% confidence interval (CI) (logistic regression) was used to investigate the relationship between fat intake indices and the risk of DN in Type 2 diabetics. *p* < 0.05 was considered statistically significant.

## 3 Results

The 138 patients were determined to be in each group. Demographic, anthropometric, and lifestyle characteristics of participants in diabetic patients with nephropathy (case group) and patients without nephropathy (control group) are shown in Table 1.

**TABLE 1:** Demographic, anthropometric and lifestyle characteristics of participants in case and control groups.

Variables	Groups, mean (SD)		*p* ^a^
	Case (*n* = 151)	Control (*n* = 158)	
Age (years)	55.1 (57.1)	57.2 (12.1)	0.146
Weight (kg)	77.3 (10.1)	77.1 (13)	0.248
BMI, (kg/m^2^)	27.39 (39.4)	27.03 (31.3)	**0.003**.
Time of diagnosis of diabetes (years)	9.16 (7.6)	7.21 (7)	**0.016**
Physical activity (Met.min/d)	1464.7 (419)	1793.08 (745.3)	**< 0.001**
Fasting insulin (mg/dL)	3.11 (59)	2.91 (0.6)	**0.006**
FBG (mg/dL)	153.41 (13.5)	140.01 (39.2)	**0.014**
HOMA‐IR	1.05 (34)	1.75 (0.3)	0.734
QUICKI	0.38 (1)	0.38 (0.1)	0.953
CRP (mg/L)	1.84 (36.0)	1.87 (0.32)	0.451
Total cholesterol (mg/dL)	198.96 (83.4)	178.82 (33)	**> 0.001**
TG (mg/dL)	241.29 (19.7)	218.86 (55)	**0.003**
HDL‐C (mg/dL)	39.18 (57.5)	40.96 (25.6)	0.404
LDL‐C (mg/dL)	128.5 (84.3)	120.6 (32.4)	**0.031**
Creatinine (mg/dL)	1.27 (47)	1.13 (47)	**0.012**
Gender			0.788
Male, *n* (%)	94 (62.3)	96 (60.8)	
Female, *n* (%)	57 (37.7)	62 (39.2)	
Type of drug use, *n* (%)			0.812
Insulin, *n* (%)	15 (9.9)	17 (10.8)	
Oral drug	136 (90.1)	141 (89.2)	
Education status, *n* (%)			0.246
Illiterate	23 (15.2)	36 (22.8)	
Less than a diploma	49 (32.5)	56 (25.4)	
Diploma	44 (29.1)	41 (25.9)	
Bachelor	32 (21.2)	24 (15.2)	
Higher than a bachelor	3 (2)	1 (0.6)	

Abbreviations: BMI, body mass index; CRP; C‐reactive protein; FBG, fasting blood glucose; HDL, high density‐lipoprotein; HOMA_IR, homeostatic model assessment‐ insulin resistance; LDL, low density‐lipoprotein; QUICKI, quantitative insulin sensitivity check index; TC, total cholesterol; TG, triglyceride.

^a^ Obtained from ANOVA for continuous variables and chi‐square for categorical variables.

Bold values represent statistically significant results: *p* < 0.05, *p* < 0.01.

There were significant differences between patients and controls in terms of variables at the time of diagnosis of diabetes; BMI, physical activity, insulin level, FBS, TC, TG, LDL‐C, and creatinine levels (*p* < 0.05).

Table 2 shows the dietary intake of the case and control groups. According to the nutritional intakes obtained from the 147‐item SFFQ, in the case group, intakes of carbohydrates, protein, fat, saturated fatty acid (SFA), monounsaturated fatty acid (MUFA), polyunsaturated fatty acid (PUFA), linoleic acid, linolenic fatty acids, cholesterol, and dietary fiber were lower compared than in the control group.

**TABLE 2:** Dietary intakes of study participants across case and control groups.

Nutrients	Groups, mean (SD)		
	Case (*n* = 151)	Control (*n* = 158)	*p*
Energy (kcal/day)	2223.9 (459.5)	2490.3 (1024.1)	0.005
Carbohydrate (g/day)	306.8 (57)	341.2 (130.6)	0.003
Protein (g/day)	83.1 (18.4)	99.6 (64.6)	0.003
Fat (g/day)	79.6 (27.2)	86.7 (35.7)	0.050
Saturated fatty acids (g/day)	20.2 (5.6)	22.3 (9.4)	0.020
Monounsaturated fatty acids (g/day)	19.7 (4.6)	23.1 (12.9)	0.003
Polyunsaturated fatty acid (g/day)	11.9 (3.3)	14.8 (6.6)	> 0.001
Linoleic acid (g/day)	9.6 (2.7)	11.9 (5.9)	> 0.001
Linolenic acid (g/day)	0.9 (0.5)	1.4 (0.8)	> 0.001
Cholesterol (g/day)	229.4 (132.6)	282.8 (177.3)	0.003
Fiber (g/day)	37.6 (11.9)	43.2 (13.7)	> 0.001

*Note:* Values are expressed as means (standard deviation [SD]). *p* values are resulted from independent sample *t*‐Test.

Dietary intake by atherogenic, thrombotic, and lipophilicity index quartiles in diabetic patients with and without nephropathy is shown in Table 3. Compared with participants in the lowest quartile of the atherosclerosis index, participants in the highest quartile of the atherosclerosis index consumed more SFAs, PUFAs, linoleic acid fatty acids, linoleic acid fatty acids, and fiber (*p* < 0.05). Dietary intakes of energy, carbohydrates, protein, fat, and cholesterol did not differ between quartiles. In terms of thrombosis index quartiles, compared with the lowest quartile, the consumption of SFAs in the highest quartile increased significantly, while the consumption of PUFA, linoleic acid fatty acid, and linoleic acid fatty acid decreased less (*p* < 0.05).

**TABLE 3:** Dietary intakes of diabetic patients with and without nephropathy among quartiles of atherogenic, thrombogenic, and lipophilic indexes.

Atherogenic index					
Variables	Q1	Q2	Q3	Q4	*p*
Energy (kcal/day)	2371.2 (542.1)	2378.9 (480.2)	2308.9 (419.9)	2399.9 (1393.8)	0.910
Saturated fatty acids (g/day)	19.6 (7.4)	20.9 (5.5)	20.5 (5.8)	23.9 (11)	0.004
Monounsaturated fatty acids (g/day)	23.1 (7.6)	21.6 (4.7)	19.9 (4.5)	21.3 (17.1)	0.251
Polyunsaturated fatty acid (g/day)	16.8 (5.7)	13.9 (3.7)	12.1 (2.7)	(7.6) 11.7	> 0.001
Linoleic acid (g/day)	12.9 (4.8)	11.1 (3.1)	9.7 (2.2)	9.4 (6.7)	> 0.001
Linolenic acid (g/day)	1.4 (0.8)	1.2 (0.6)	1.1 (0.4)	0.9 (0.7)	> 0.001
Fiber (g/day)	43.1 (13.2)	42.4 (15.2)	38.6 (11.6)	37.9 (11.7)	0.029
Thrombogenic index					
Energy (kcal/day)	2364.3 (528.5)	2351.7 (515.1)	2398.6 (451.6)	2343.13 (1377.5)	0.976
Saturated fatty acids (g/day)	19.7 (7.2)	21.4 (6.5)	20.7 (5.6)	23.2 (10.9)	0.042
Monounsaturated fatty acids (g/day)	22.6 (7.5)	21.8 (5.3)	20.4 (4.4)	21 (17.7)	0.540
Polyunsaturated fatty acid (g/day)	16.5 (5.5)	13.5 (3.6)	12.4 (2.2)	11.2 (7.6)	> 0.001
Linoleic acid (g/day)	13.5 (4.6)	10.8 (3.1)	9.8 (1.8)	8.9 (6.7)	> 0.001
Linolenic acid (g/day)	1.6 (0.8)	1.2 (0.5)	0.9 (0.3)	0.8 (0.7)	> 0.001
Fiber (g/day)	43.2 (12.8)	40.4 (14.7)	40.1 (12.8)	38.4 (11.8)	0.142
Lipophilic index					
Energy (kcal/day)	2352.8 (507.7)	2384.9 (526.4)	33.1 (419.6)	2389.9 (1390.9)	0.965
Saturated fatty acids (g/day)	19 (6.8)	21.7 (6.5)	20.7 (5.1)	23.6 (11.1)	0.003
Monounsaturated fatty acids (g/day)	21.9 (7.5)	22.1 (5.4)	20.3 (4.2)	21.5 (17.1)	0.654
Polyunsaturated fatty acid (g/day)	16.3 (5.6)	13.8 (3.6)	12.1 (2.2)	11.4 (7.6)	> 0.001
Linoleic acid (g/day)	13.3 (4.7)	11.2 (2.9)	9.6 (1.8)	9 (6.7)	> 0.001
Linolenic acid (g/day)	1.6 (0.8)	1.2 (0.5)	0.9 (0.3)	0.8 (0.7)	> 0.001
Fiber (g/day)	43.4 (13.7)	41.5 (15.2)	38.5 (10.5)	38.5 (12.2)	0.045

*Note:* Values are expressed as means (standard deviation [SD]). *p* value results from ANOVA.

In terms of comparison of participants' dietary intake stratified by lipophilic index quartiles, cholesterol, and SFA, intake increased significantly in the highest quartile compared to the lowest quartile. The intake of PUFAs, linoleic fatty acids, linolenic fatty acids, and fiber, decreased significantly.

Table 4 shows the differences between atherogenic, thrombotic, and lipophilic index quartiles in our diabetic nephropathy model.

**TABLE 4:** Odds ratio (OR) and 95% confidence interval (CI) for diabetic nephropathy (DN) based on quartiles of atherogenic, thrombogenic, and lipophilic indexes.

Variables	Quartiles of atherogenic, thrombogenic, and lipophilic indexes				*p* for trend
	Q1	Q2	Q3	Q4	
Atherogenic index					
Case/total (*n*)	24.77	33.77	47.78	47.77	—
Crude model^a^	1.00 (Ref)	1.82 (0.93–3.56)	3.54 (1.81–6.93)	3.66 (1.86–7.19)	< 0.001
Model 1^b^	1.00 (Ref)	1.98 (0.98–3.97)	3.50 (1.74–7.04)	3.53 (1.75–7.12)	< 0.001
Model 2^c^	1.00 (Ref)	2.14 (0.99–4.6))	3.39 (1.56–7.37)	3.49 (1.64–7.41)	< 0.001
Thrombogenic index					
Case/total (*n*)	22.77	36.77	44.78	49.77	—
Crude model^a^	1.00 (Ref)	2.43 (1.23–4.87)	3.42 (1.74–6.74)	4.66 (2.34–9.29)	< 0.001
Model 1^b^	1.00 (Ref)	2.52 (1.24–5.12)	3.59 (1.78–7.24)	4.14 (2.03–8.44)	< 0.001
Model 2^c^	1.00 (Ref)	2.05 (0.94–4.46)	3.21 (1.49–6.91)	4.03 (1.86–8.72)	< 0.001
Lipophilic index					
Case/total (*n*)	25.77	32.77	46.78	48.77	—
Crude model^a^	1.00 (Ref)	1.58 (0.81–3.08)	3.25 (1.67–6.35)	3.64 (1.85–7.13)	< 0.001
Model 1^b^	1.00 (Ref)	1.63 (0.81–3.25)	3.29 (1.65–6.58)	3.34 (1.66–6.72)	< 0.001
Model 2^c^	1.00 (Ref)	1.46 (0.68–3.15)	2.84 (1.34–6.01)	3.50 (1.62–7.52)	< 0.001

^a^ Binary logistic regression was used to obtain OR and 95% CI. The overall trend of OR across increasing quartiles was examined by considering the median score in eachcategory as a continuous variable.

^b^ Model 1: adjusted for age, BMI and energy.

^c^ Model 2: adjusted for Model 1 and physical activity, fasting glucose, fasting insulin, C‐reactive protein, total cholesterol, triglyceride, high density‐lipoprotein, low density‐lipoprotein, and Creatinine.

In the crude model, diabetic patients in the highest quartile of the atherogenic index had a higher risk of nephropathy than diabetic patients in the lowest quartile (OR: 3.66, 95% CI: 1.86–7.19, *p* trend < 0.001). In the model adjusted for potential confounding factors such as age, BMI, and energy, a significant association was found between nephropathy and atherosclerosis index (OR: 3.53, 95% CI: 1.75–7.12, *p* trend < 0.001). This association remained significant after adjusting for confounding variables (physical activity, fasting glucose, fasting insulin levels, and lipids including TC, TG, LDL‐C, HDL‐C, creatinine, and CRP) in the second model. In this way, the findings showed that the odds' ratio of DN in T2DM patients who were in the highest quartile of atherogenic index is almost three times higher than in T2DM patients who are in the lowest quartile of atherogenic index (OR: 3.49, 95% CI: 1.65–7.41, *p* trend < 0.001).

We found a positive association between the risk of nephropathy in diabetic patients in the highest quartile of thrombotic index compared with patients in the lowest quartile of the crude pattern (OR: 4.66, 95% CI: 2.34–9.29, *p* trend < 0.001), Model 1 adjusted for age, BMI and energy (OR: 4.14, 95% CI: 2.03–8.44, *p*_trend_ < 0.001), and Model 2 adjusted for physical activity, fasting blood glucose, fasting insulin level, lipid profile including cholesterol, TG, LDL, HDL, creatinine level, and CRP (OR: 4.03, 95% CI: 1.86–8.72, *p*_trend_ < 0.001).

In the crude model, in comparison to the lowest quartile, the highest quartile of the lipophilic index was significantly associated with a 3.64‐unit greater risk of nephropathy (OR: 3.64, 95% CI: 1.85–7.13, *p*_trend_ < 0.001).

After adjusting confounders in model 1, for age, BMI, and energy, and model 2, for physical activity, fasting blood glucose, fasting insulin level, and lipid profile including cholesterol, TG, LDL, HDL, creatinine level, and CRP, we observed a significant relationship between increased risk of nephropathy in diabetic patients with the highest quartile of the thrombogenic index compared to diabetic patients in the lowest quartile (OR: 3.34, 95% CI: 1.66–6.72, *p* trend < 0.001) and (OR: 3.50, 95% CI: 1.62–7.52, *p* trend < 0.001), respectively.

## 4 Discussion

This study examined the association between atherogenic (IA), thrombogenic (IT), and lipophilic (LT) indices—as fat intake indicators—and the risk of nephropathy in T2DM patients, after adjusting for glycemic index, lipid profile, and CRP levels. This study found T2DM patients in the highest quartile of atherogenic, thrombogenic, and lipophilic indices had a greater likelihood of developing DN. Also, based on findings, higher values of IA, IT, and LT were associated with a higher probability of DN in T2DM patients. In addition, dietary MUFA, PUFA and SFA fatty acids were associated with atherogenic, thrombogenic and lipophilic indices.

According to epidemiological studies, the prevalence of DN is increasing (Zhou et al. 2018). The vascular, metabolic and inflammatory disorders caused by diabetes mellitus are often associated with progressive albuminuria, which can lead to kidney damage and DN (Shapiro et al. 2011). Early DN is characterized by microalbuminuria, progressing albuminuria, and elevated serum creatinine levels, ultimately resulting in kidney failure (Weng et al. 2016). The pathogenesis of DN remains unclear; however, studies indicate several molecular pathways involved in its development, including the activation of polyol and protein kinase C pathways, oxidative stress, generation of advanced glycation end products (AGEs), and inflammation (Tripathi and Yadav 2013; Wada and Makino 2013). In fact, the infiltration of inflammatory cells into the glomerulus is crucial for the development of DN (Zhou et al. 2018). A meta‐analysis found that serum TNF‐α levels were significantly elevated in T2DM patients and even higher in those with DN, indicating that DN increases inflammatory burden (Chen et al. 2017).

Our findings demonstrate the relationship between IA, IT and LI and the risk of DN in T2DM patients. The Atherogenic index shows the relationship between SFA content and UFAs (Moussavi Javardi et al. 2020). In this study, diabetics in the highest quartile of the IA were 3.5 times more likely to develop nephropathy than diabetics in the lowest quartile. This study showed Intake of PUFA, linoleic acid, and linoleic acid in the fourth quartile was lower than in the first quartile. In other words, based on the results of this study, patients with higher IA value or in the higher quartile of IA have lower PUFA intake and show higher DN progression.

Various studies have investigated the relationship between FAs and diabetes and DN (de Boer et al. 2019; Lin et al. 2022; Liu et al. 2023, 2024). This finding is consistent with a previous study on 3287 participants with T2DM in the National Health and Nutrition Examination Survey (NHANES) that suggested increased PUFA intake may slow DKD progression (Liu et al. 2023). Based on an animal study, chronic hyperglycemia in diabetes patients can cause low‐grade inflammation, but improving dietary fat quality—by decreasing total fat and SFA while increasing UFAs—can enhance inflammation and metabolic syndromes (Hwang et al. 2022). In addition, Dos Santos et al. (2018) suggested the low intake of alpha‐linolenic acid and linoleic acid is related to the development of DN. Thus, it seems the n‐6 fatty acids seem to have a positive effect on DN (Nakamura et al. 2023). These findings are consistent with the results of the current study.

In addition, the studies indicate an inverse relationship between dietary intake of EPA and DHA and urinary albumin excretion (UAE) in diabetes patients (de Boer et al. 2019; Lin et al. 2022). This suggests that n‐3 fatty acids like EPA and DHA may effectively reduce UAE and help prevent DN (Han et al. 2016). Lee et al. (2010) found an inverse relationship between dietary intake of EPA + DHA and the degree of the UAE in Type 1 diabetes. Han et al. (2016) had shown that T2DM patients with hypertriglyceridemia taking n‐3 FAs experienced reduced UAE and a halt in renal function decline. The possible mechanisms of the effect of long‐chain PUFA on reducing inflammation are as fallows; EPA reduces insulin resistance and adipose tissue inflammation by enhancing adiponectin levels and decreasing pro‐inflammatory cytokines (D'Angelo et al. 2020). In addition, Docosahexaenoic acid may reduce fractalkine expression and secretion by inhibiting the tumor necrosis factor‐α signaling pathway in ND patients, thereby alleviating kidney inflammation and oxidative stress and delaying disease progression (Zhou et al. 2018). Also, swapping SFA for UFA reduces inflammation, likely because the body stores less fat from UFAs than from SFAs at the same caloric intake (Rosqvist et al. 2019). Anyway, further investigation into n‐6/n‐3 ratios is needed for kidney protection (Liu et al. 2024). In the present study, n‐3 fatty acids were not investigated separately. It is necessary to assess n‐3 and n‐6 acids separately in future studies.

An association between diet LI and plasma triglyceride concentrations was found. A higher LI seems to be related to a decrease in the fluidity of fatty acids. This leads to the accumulation of triglycerides and thus increases insulin resistance, and the risk of cardiovascular disease (Toledo et al. 2013), that is also associated with inflammatory conditions (Sincer, Gunes, and Mansiroglu 2020). The results of this study showed that diabetics in the highest quartile of the lipophilicity index were 3.5 times more likely to develop nephropathy than diabetics in the lower quartile of the lipophilicity index. Additionally, the intake of SFAs in the fourth quarter was higher than in the first quarter, while the intake of PUFAs, linolenic acid, and linolenic acid in the fourth quarter was lower than in the first quarter.

Early intervention was crucial for achieving remission of DN (Lin et al. 2022). Although several studies have investigated the relationship between kidney disease and fatty acids DN (Lin et al. 2022; Nakamura et al. 2017, 2023), the results do not necessarily show a significant relationship, and this issue remains controversial (Nakamura et al. 2023). For example, in a study on T1DM patients with increased albuminuria, urinary albumin excretion was raised by 58% after consuming more linoleic acid (LA) (Dos Santos et al. 2018). The results for a healthy population are inconsistent. In a study on 2600 healthy adults, the increasing n‐6 intake resulted in an increase in the incidence of chronic kidney disease was observed (Gopinath et al. 2011). Also, it was suggested that n‐6 fatty acids may offer greater nephroprotection in certain pathological conditions (Nakamura et al. 2023). Therefore, the effect of n‐6 on kidney disease is controversial. Differences in study design such as different sample sizes, differences between selected populations, and other factors can be considered as an explanation for the difference between our results and the mentioned studies. The impact of amino acid (AA) metabolites in the kidney has been suggested as one of the reasons to be various results in FA intake balance and DN (Lucas et al. 2019). Prostaglandins (PGs) are metabolized from AA. These compounds, like PGE2, have a diuretic effect due to inhibition of sodium reabsorption and antagonism with antidiuretic hormone (ADH) (Baker and Perazella 2020; Lucas et al. 2019). Therefore, it is suggested n‐6 fatty acids may offer greater Reno protection than n‐3 fatty acids in certain pathological conditions (Nakamura et al. 2023).

## 5 Strengths and Limitations of the Study

This study has several strengths. To our knowledge, ours is the first case–controll study to evaluate the relationship between fat intake indices and the risk of DN. Moreover, a valid FFQ and physical activity questionnaire were used. Additionally, in this study, all confounding factors including glycemic index, lipid profile, and CRP concentration that might affect the results were considered and adjusted in the statistical analysis. Despite these benefits, some limitations should be noted. Considering that this study was a cross‐sectional/case–control study, the participants were not followed up in terms of assessing their dietary intake over time and their health status. While using an accurate and reliable FFQ to estimate nutritional intake, there is always the risk of measurement errors. Moreover, most case–control tests can be used to determine the cause‐and‐effect relationship, but it cannot be absolutely proved.

## 6 Conclusion

We found that diabetic patients in the highest quartile of the atherogenic, thrombogenic, and lipophilic indices had a higher chance of developing nephropathy than those in the lowest quartile, even after adjusting for confounding variables such as glycemic control, lipid profile, and inflammation. According to our findings, the risk of DN in T2DM patients who have higher atherogenic, thrombogenic and lipophilic indices is more than T2DM patients who have lower atherogenic, thrombogenic and lipophilic indices. In addition, our study showed that the composition of dietary macronutrients and especially the types of MUFA, PUFA and SFA fatty acids may be associated with atherogenic, thrombogenic and lipophilic indices. Therefore, it can be said that there is a correlation between the composition of dietary macronutrients and especially the MUFA, PUFA and SFA fatty acids with the occurrence of DN in patients with T2DM. Therefore, it may be possible to change the composition of dietary macronutrients in people with T2DM, as well as to improve BMI, to improve atherogenic, thrombogenic and lipophilic indices, thus reducing the chance of diabetic nephropathy. However, more research is needed to understand this relationship.

## 7 Practical Applications

Based on our findings, we recommend that patients with high atherogenic, thrombogenic, and lipophilic indices take specific steps to reduce their risk of diabetic nephropathy. These steps include reducing your intake of saturated and trans fats, increasing your intake of polyunsaturated and monounsaturated fatty acids, and eating high‐fiber foods such as fruits, vegetables, and whole grains. We also recommend that patients limit their intake of processed foods, sugary drinks, and red and processed meats that are high in saturated fats and added sugars.

## Author Contributions

**Behnood Abbasi:** conceptualization (equal), data curation (equal), formal analysis (equal), funding acquisition (equal), investigation (equal), methodology (equal), project administration (lead), resources (equal), software (equal), supervision (lead), validation (equal), visualization (equal), writing – original draft (equal), writing – review and editing (lead). **Elham Ghanbarzadeh:** conceptualization (equal), data curation (equal), formal analysis (equal), funding acquisition (equal), investigation (equal), methodology (equal), project administration (supporting), resources (equal), software (equal), validation (equal), visualization (equal), writing – original draft (equal), writing – review and editing (supporting). **Bita Panahizadeh:** formal analysis (equal), investigation (supporting), methodology (equal), software (equal), validation (supporting), visualization (supporting).

## Ethics Statement

The Ethics Committee of Mazandaran University of Medical Sciences approved this study (IR.IAU.SRB.REC.1398.091). The purpose of the study was fully explained to all participants, and written informed consent was obtained from all participants.

## Consent

The authors have nothing to report.

## Conflicts of Interest

The authors declare no conflicts of interest.

## Data Availability

The data that support the findings of this study are available on request from the corresponding author. The data are not publicly available due to privacy or ethical restrictions.
